# ReClassification of Patients with Ambiguous CA125 for Optimised Pre-Surgical Triage

**DOI:** 10.3390/diagnostics14070671

**Published:** 2024-03-22

**Authors:** Andrew N. Stephens, Simon J. Hobbs, Sung-Woog Kang, Martin K. Oehler, Tom W. Jobling, Richard Allman

**Affiliations:** 1 Hudson Institute of Medical Research, Clayton 3168, Australia; kansu353@gmail.com; 2 Department of Molecular and Translational Sciences, Monash University, Clayton 3168, Australia; 3 Cleo Diagnostics Ltd., Melbourne 3000, Australia; simon.hobbs@cleodx.com (S.J.H.); richard.allman@cleodx.com (R.A.); 4 Department of Gynecological Oncology, Royal Adelaide Hospital, Adelaide 5000, Australia; oehler.mk@gmail.com; 5 Robinson Institute, University of Adelaide, Adelaide 5000, Australia; 6 Department of Gynecological Oncology, Monash Medical Centre, Bentleigh East 3165, Australia; tjobling@bigpond.net.au

**Keywords:** ovarian, cancer, CXCL10, biomarker, diagnostic, triage, malignancy, benign

## Abstract

Pre-surgical clinical assessment of an adnexal mass is a complex process, and ideally requires accurate and rapid identification of disease status. Gold standard biomarker CA125 is extensively used off-label for this purpose; however its performance is typically inadequate, particularly for the detection of early stage disease and discrimination between benign versus malignant status. We recently described a multi-marker panel (MMP) and associated risk index for the differentiation of benign from malignant ovarian disease. In this study we applied a net reclassification approach to assess the use of MMP index to rescue those cases where low CA125 incorrectly excludes cancer diagnoses, or where benign disease is incorrectly assessed as “high risk” due to elevated CA125. Reclassification of such patients is of significant value to assist in the timely and accurate referral for patients where CA125 titer is uninformative.

## 1. Introduction

Vague symptoms and a lack of accurate diagnostic approaches are key contributors to the high five-year mortality rate for ovarian cancer patients, with 314,000 new cases and 207,000 deaths worldwide in 2020 [1]. Diagnosis at an early stage, prior to extra-ovarian involvement, is associated with >90% five-year survival; hence population-based screening is widely believed to be crucial to achieve an overall reduction in ovarian cancer mortality. Despite significant research efforts [2], there remains no effective approach for screening-based early identification of ovarian cancers.

The “gold-standard” ovarian cancer biomarker cancer antigen 125 (CA125) is FDA-approved for monitoring therapeutic responses and disease recurrence. Broadest clinical use of CA125 actually occurs off-label, however, in combination with transvaginal ultrasound (TVU) to determine the risk of malignancy in patients who present with an adnexal mass. Whilst international guidelines vary regarding the clinical work-up of an adnexal mass [3,4,5], a threshold of CA125 > 35 U/mL is considered “elevated” in post-menopausal women and is the trigger for referral to a gynaecological oncologist. However, current clinical guidelines do not specify what constitutes “elevated” CA125 in pre-menopausal women, and ranges from 67 U/mL to 250 U/mL have been recommended [3,4,6].

CA125 is an imperfect marker for ovarian cancer. Around 20% of ovarian cancers do not express CA125 [7,8], and patients with early-stage disease often do not have elevated CA125 serum titres [9]. Combined with the generally asymptomatic presentation of ovarian cancers, false-negative CA125 results are common and complicate the appropriate referral of patients to a gynaecological oncology specialist for primary surgery [10,11]. Conversely, CA125 may be abnormally elevated in non-malignant conditions (e.g., endometriosis, fibroids, adenomyosis, pelvic infection and pelvic inflammatory disease [3]) leading to high false-positive detection rates. Unsurprisingly, post-surgical diagnoses of benign disease out-number malignancies by ~9:1 [4,12]. This is a particularly important issue for pre-menopausal women, who generally have higher serum CA125 titres [7] and a significantly lower incidence of ovarian cancer than post-menopausal women [5]. False-positive detection in this group can trigger referral to a gynaecological oncologist for surgery, where the primary concern is removal of suspicious tissues and not preservation of fertility or ovarian function [13,14,15,16].

The early identification and differentiation of benign from malignant adnexal masses is paramount in determining outcomes for patients. It is well-established that immediate referral to a gynaecological oncology specialist results in the best outcomes for cancer patients [11,17,18,19,20]. Conversely, early identification of non-malignant disease can guide the use of less invasive surgical interventions (for example, laparoscopy) to achieve optimal patient outcomes and do not need to be performed by an oncology specialist [21]. International guidelines for the management of adnexal masses stipulate the use of radiological imaging (typically transvaginal ultrasound) to better characterise adnexal masses and guide referral for secondary evaluation [22]. Depending on the guideline followed, imaging may be performed prior to, in conjunction with, or after biomarker testing for CA125 [3,4,5,23]. The nature and order of testing are largely dictated by cost and resource availability; for example, even though the diagnostic efficacy of CA125 is low [24], it remains recommended under the National Institute for Health and Care Excellence (NICE) guidelines in England and Wales for first-line investigation as part of the “2-week wait suspected cancer pathway” [5,25]. Recent evaluation of this approach suggests that CA125 is an inefficient method of referral, as most patients referred for imaging based on CA125 titres do not have cancer [25]. Thus, testing modalities and the order in which they are applied can have implications for the detection of cancers, as well as the exclusion (and minimisation of the psychological impacts) of women with benign adnexal masses.

In the United States, imaging typically precedes biomarker evaluation [4]. With the aid of commonly used risk tools such as Risk of Malignancy Index (RMI), International Ovarian Tumour Analysis (IOTA) or Assessment of Different NEoplasias in the adneXa (ADNEX) scoring, high quality ultrasound imaging can correctly triage up to ~80% of cases with short turnaround times; these patients then undergo further testing (including biomarkers) and surgical referral as warranted [25,26,27]. For the remaining 20% of patients that return an “indeterminate” result, however, it is paramount that they are appropriately referred for evaluation within as short a timeframe as possible (typically 24 h). Interestingly, the measurement of CA125 provides little additional efficacy to this process [27,28]. Exclusion of false positive cases is therefore an important target for improved biomarker testing in addition to the capture of as many cancers as possible during the pre-surgical evaluation process. To have clinical relevance for pre-surgical triage, a biomarker-based test must therefore provide (i) clear additive efficacy to imaging in those cases where an indeterminate result is obtained; (ii) differentiation benign from malignant disease with high accuracy, particularly for early stage malignancies; (iii) similar efficacy in pre- and post-menopausal women; and (iv) short turnaround times for testing following receipt of an indeterminate ultrasound finding.

In the absence of consistent guidelines for CA125 use, research has focused on the development of multi-modal biomarker panels to improve surgical triage and reduce the incidence of false negative or positive CA125 test results. In a recent study we described the use of a Multi-Marker Panel (MMP) index comprising CA125, Human Epididymis Protein 4 (HE4), Interleukin-6 (IL6) and the C-X-C motif chemokine ligand 10 (CXCL10) active ratio [29] to indicate the risk of malignancy [30]. The panel provided a substantial improvement in accuracy over CA125 for the classification of malignant samples against a background of benign disease [30].

Of particular interest within this cohort are those patients for whom CA125 failed to provide an appropriate classification; i.e., cancer cases missed because of a low CA125 serum titre, and benign cases where elevated CA125 resulted in mis-classification and triage to a gynaecological oncologist. We have applied a risk reclassification approach [31] to explore whether MMP index can add value over existing testing, specifically in cases where CA125 results fall outside accepted clinical thresholds.

## 2. Materials and Methods

### 2.1. Patient Cohort

The patient cohort was assembled from a multi-centre retrospective collection conducted between 2007 and 2021, and included 334 patients as previously described [30]. Ethical approval was obtained from the Southern Health Human Research Ethics Committee (HREC #06032C and #02031B), and all participants provided prior informed written consent. Summarised details regarding confirmed diagnoses and CA125 titres (the focus for this study) are provided in Table 1. In brief, samples were retrospectively collected from anaesthetised, chemo-naïve patients referred for surgery for suspected gynaecological malignancy. Post-surgical diagnoses were confirmed from hospital records. Imaging data were reviewed and scored by a gynaecological oncology specialist. A full description of the cohort, including comprehensive pathology, was provided previously [30].

### 2.2. Risk Prediction Models

CA125 serum tires, accompanying post-surgical diagnoses and scoring were as described in [30]. For post-menopausal women, a cut-off of 35 U/mL was used according to international guidelines [4]. For pre-menopausal women three different cut-off values from 67 U/mL to 250 U/mL [3,4,6] were individually assessed. Net re-classification index (NRI) was calculated as described in [32], using a binary re-classification into high risk (cancer) or low risk (benign) groups. Re-classification scores and +/−95% confidence intervals were calculated as percentages of the total number of cases or controls, respectively.

Linear regression models, the calculation of MMP Index and all other statistical analyses were conducted as previously reported [30]. In brief, a diagnostic algorithm for the multi-marker panel was developed using a multivariate logistic regression model and applied for the differentiation of benign from malignant samples. A scoring cut point of 3.684 was determined that provided 95% sensitivity and 95% specificity for differentiation between samples of benign and malignant origin [30]. Classifications according to the multi marker panel were compared directly against CA125 titre. All other statistical analyses, including Student’s *t*-tests and the generation of x–y plots, were performed using GraphPad Prism v10.0.3 (275) (Boston, MA, USA).

## 3. Results

### 3.1. Use of MMP Index Successfully Rescues Samples Mis-Classified by CA125

A summary of relevant characteristics of the cohort is provided in Table 1. Malignancy was diagnosed more commonly in the post-menopausal group and was generally late stage (Table 1). Reported CA125 titres were not significantly different between pre- and post-menopausal women grouped according to disease stage, although pre-menopausal women diagnosed with stage I cancer trended towards higher median CA125 values compared to post-menopausal women diagnosed at the same stage (*p* = 0.059; Student’s *t*-test). Within the pre-menopausal cohort, CA125 was significantly elevated in cancers relative to benign cases (stage I vs. benign, *p* < 0.0001; stages II–IV vs. benign, *p* < 0.0001) as well as between cancer stages (stage I vs. stages II–IV, *p* = 0.03). Similar findings were observed within the post-menopausal cohort (stage I vs. benign, *p* = 0.0003; stages II–IV vs. benign, *p* < 0.0001; stage I vs. stages II–IV, *p* = 0.00).

**Figure 1:**
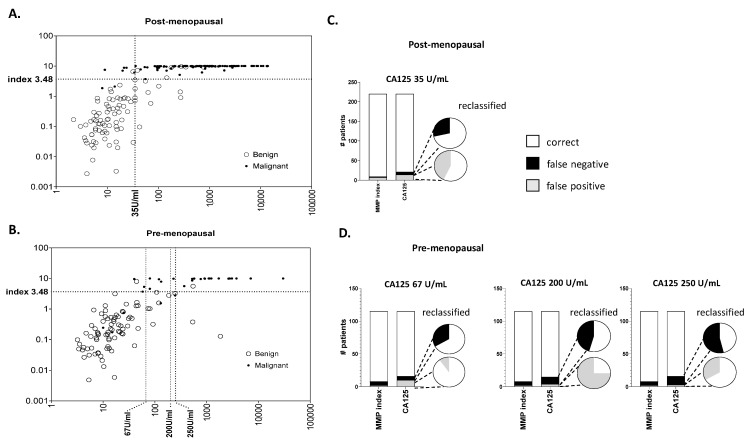
Distributions and classification error rates amongst patients with false negative or false positive CA125 outcomes. (**A**,**B**) Distribution of patient samples scored according to CA125 or Multi Marker Panel (MMP) index. Dotted lines indicate cut-off values according to the literature for post- or pre-menopausal women, respectively. Confirmed diagnosis (benign or malignant) for each patient is indicated. (**C**,**D**) Comparison of mis-classifications at different thresholds for CA125 (35 U/mL, 67 U/mL, 200 U/mL, or 250 U/mL) versus the MMP index amongst total cases. Bar graphs illustrate the numbers of false positive (cancer < CA125 threshold—grey) or false negative (benign > CA125 threshold—black) findings in the cohort at each threshold. Pie charts show the proportion of cases corrected following re-classification by MMP index when CA125 provided an incorrect result. Grey or black shading indicates those false positive or negative cases, respectively, remaining after reclassification.

The distribution of sample pathology against cut-off thresholds for MMP index vs. CA125 in each of the post- and pre-menopausal cohorts is shown in Figure 1A,B, respectively. For post-menopausal patients a single threshold of 35 U/mL was used, as specified by international guidelines [4]. For pre-menopausal patients, three different threshold values at 67 U/mL [6], 200 U/mL [4] and 250 U/mL [3] were assessed. As expected, increasing the CA125 cut-off threshold in the pre-menopausal cohort had clear influences on the sensitivity and specificity of testing by CA125 (Figure 1A,B). In all cases, misclassifications across the entire cohort were higher using CA125 at each tested threshold compared to MMP index (Figure 1C,D). For both post-menopausal (Figure 1C) and pre-menopausal (Figure 1D) women, the MMP index correctly re-classified most false negative and false positive cases suggested using CA125. Overall there was a reduction of between ~2- to 5-fold in mis-classifications when samples were re-evaluated using MMP index.

The converse situation, where MMP index mis-classified a patient that CA125 correctly classified, was previously reported [30] and was only observed in 3 cases. Amongst these was one pre-menopausal patient (age 41 years, CA125 at 246 U/mL) with a stage I grade 3 mixed endometroid/clear cell tumour; and one post-menopausal patient (age 87 years, CA125 at 32 U/mL) with benign cystadenofibroma. Interestingly, the third mis-classification was of a patient with ovarian endometriosis and a CA125 titre at 44.2 U/mL; this patient self-reported as pre-menopausal but was aged 51 years at the time of diagnosis, suggesting menopausal status (and thus classification by CA125) may not have been accurate. Overall, very few patients with accurate CA125 classifications were mis-classified by MMP index.

### 3.2. Net Re-Classification Analysis Improves Case/Control Assignment at Clinical Thresholds

Net reclassification risk analysis was used to evaluate the efficacy of re-assignment of patients into low risk (benign) or high-risk (malignant) groups by MMP index compared to CA125 score. In each case the net re-classification index (NRI) was calculated using a single MMP index score of 3.48 [30] against CA125 at 35 U/mL (post-menopausal) or 67 U/mL, 200 U/mL, and 250 U/mL (pre-menopausal).

NRI showed significantly improved performance of the MMP index compared to CA125 in post-menopausal women at 35 U/mL and in pre-menopausal women at 200 U/mL and 250 U/mL; when a CA125 threshold of 67 U/mL was used, a similar but non-significant change was also observed (Table 2). Of particular interest, the greatest improvements in the re-classification of cancer cases were achieved for pre-menopausal patients in whom “missed” cancers (i.e., those with CA125 below the threshold) were correctly re-assigned using the MMP index (Table 2). Similarly, an improved re-assignment of controls as lower risk (i.e., benign cases with CA125 above the threshold) was also observed; interestingly, the most substantial change was made at the 67 U/mL threshold despite the overall NRI failing to reach significance (Table 2). The data indicate that the MMP index provided a substantial advantage for the re-classification of samples with ambiguous CA125 titres, particularly in the pre-menopausal group.

#### 3.2.1. Re-Classification of Post-Menopausal Patients

International guidelines stipulate a CA125 threshold of 35 U/mL as the trigger for referral of post-menopausal patients to a gynaecological oncologist. Amongst 7 cancers mis-classified by CA125 (false negative rate 8.8%), 5 were correctly re-classified as cancers using MMP index (Table 3 and Supplementary Data S1). All re-assigned samples were of high grade serous pathology (grades 2–3), including one stage I cancer (CA125 = 9) and four stage III cancers (CA125 range 24–34). Two low grade cancers remained incorrectly assigned in either case. Within the same cohort, 6 out of a total of 14 benign cases with CA125 > 35 U/mL (false positive rate 10.1%) were correctly re-assigned using the MMP index (Table 3). Overall there were ~71% and ~57% reduction in false positive and false negative detections, respectively, in post-menopausal women.

#### 3.2.2. Reclassification of Pre-Menopausal Patients

No explicit guidelines for CA125 threshold in pre-menopausal women are stipulated in international guidelines, beyond a suggestion that referral to a gynaecological oncologist should be considered when CA125 > 200 U/mL (or >250 U/mL) [3,4]. Classification was therefore assessed according to previously suggested CA125 thresholds of 67 U/mL [6], 200 U/mL [4] and 250 U/mL [3].

As anticipated an increasing number of cancers were correctly re-classified by MMP index as the CA125 threshold was raised (Table 3). Amongst 13 mis-classified cancers with CA125 below 250 U/mL, 7 were correctly re-classified using the MMP index (including 4 stage Iancers). Similarly, 6 and 4 cancers were re-classified when the lower CA125 thresholds of 200 U/mL or 67 U/mL were evaluated, respectively; including 3 or 1 stage I cancers in each case. Improved re-assignment of benign cases with elevated CA125 was also observed; the greatest change occurred at a CA125 threshold of 67 U/mL, where 9/10 patients were correctly re-classified as low-risk compared to CA125 (Table 3). Amongst the entire cohort (including both pre- and post-menopausal), only a single patient with benign fibroma was not reassigned after reclassification using MMP index.

## 4. Discussion

False positive detection of benign disease by CA125 outnumbers malignancy by approximately 9:1 [4,12] and is a significant issue for triage of patients who present with an adnexal mass. An elevated CA125 test has significant psychological burden for patients, including increased anxiety and psychological morbidities, whilst they wait for a surgical diagnosis to be made [2,33,34]. The development of more accurate testing is thus desirable to ensure that patients with non-malignant disease can be provided an alternate pathway; particularly for pre-menopausal women, where fertility preservation strategies require early specialist input [4,13,14,15].

Due to multiple co-morbidities associated with oophorectomies (e.g., increased risk of heart disease, osteoporosis and some cancers) [35,36,37], current ACOG guidelines suggest a conservative surgical approach for the management of adnexal mass and the use of laparoscopic techniques where possible [4]. Of particular interest therefore was the re-classification of “false positive” patients with elevated CA125 who were subsequently diagnosed with benign disease. Reclassification using the MMP index excluded 8/14 (57%) of post-menopausal patients and up to 9/10 (90%) of pre-menopausal patients with elevated CA125 levels but benign adnexal mass. Whilst a CA125 threshold of 35 U/mL is well-established in post-menopausal women, there is no similar approved CA125 cutoff used in pre-menopausal women. Prior ACOG guidelines suggested a working threshold of 200 U/mL as the trigger for referral of pre-menopausal patients to a gynaecological oncologist [38]. However, the most recent advice suggests that “care providers should integrate the CA 125 level with other clinical factors in judging the need for consultation” [4].

Improved elimination of false-positive CA125 results has particular relevance in pre-menopausal women, where cancer diagnoses are typically rare. In this cohort initial clinical workup is focused on determining the risk of malignancy, and biomarker measurements are recommended when imaging provides an indeterminate result [39]. Analysis of in-patient hospitalisations in the US over a 7 year period identified ~428,000 pre-menopausal women admitted for adnexal masses [40]. Whilst ~54% of these women underwent surgery, ~27% of patients (~116,000 women) were excluded following further clinical work-up. Administration of an accurate biomarker test early in the clinical workflow could therefore substantially decrease the requirement for hospital admissions and reduce overall burden on the healthcare system. Our data suggest that MMP index can provide a superior pre-surgical measurement to correctly identify patients in whom CA125 proves inadequate, and who may benefit from primary referral to a gynaecologist rather than an oncology specialist.

Arguably more crucial is the requirement for “rescue” of those cancer patients in whom a low CA125 test outcome provides a false negative indication. This is a particularly important consideration in the pre-surgical setting, where rapid identification and triage to a gynaecological oncology surgeon is critical to achieve greatest benefit for patients [18,41,42]. Reclassification using MMP index captured up to 71% of post-menopausal and between 33–67% of pre-menopausal “low CA125” cancers, respectively, substantially reducing overall false negative rates. Dearking previously suggested a CA125 threshold of 67 U/mL for improved sensitivity of cancer detection, at which specificity was not changed [6]. A recent study by Dunton compared the performance of OVA1^TM^ against this benchmark, and demonstrated an improvement of ~50% for identification of “low CA125” cancers in premenopausal women [43]. Whilst in a substantially smaller dataset, MMP index identified >60% of “low risk” cancers in both pre- and post-menopausal groups at the same thresholds, suggesting performance improvement over CA125 and OVA1^TM^.

Early stage diagnosis of ovarian cancers, combined with rapid referral to an oncologist, is critical to optimize patient outcomes and overall survival. For patients with CA125 below the prescribed threshold, or if elevated but without concordant ultrasound findings, current guidelines suggest a “watch and wait” strategy [4,5]. Uncertainty around a potential ovarian cancer diagnosis can delay referral, potentially reducing the likelihood of successful treatment. Amongst stage I cancers mis-classified due to low CA125 in this study, reclassification analysis identified up to 6/9 (dependent on CA125 threshold used) for a total of 66% improvement over CA125. This included 1/2 (50%) in the post-menopausal group, and up to 5/7 (71%) in the pre-menopausal group, suggesting a significant improvement in capability for early stage detection.

**Figure 2:**
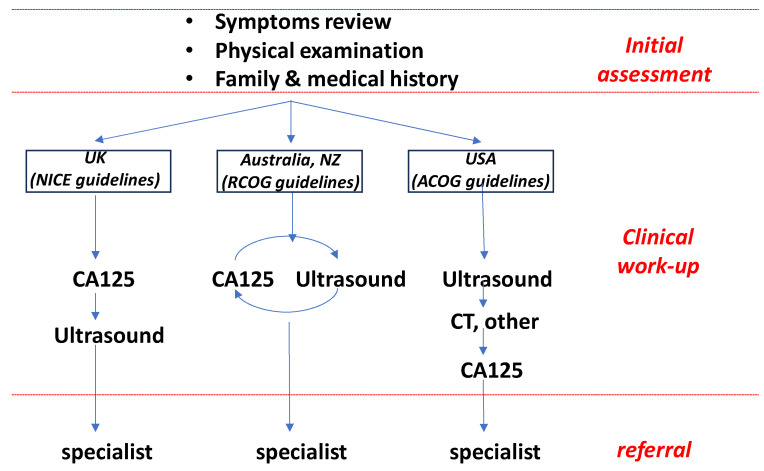
Typical clinical workflow for diagnosis and referral of patients with adnexal mass. Dependent on geographical region and guidelines adopted patients experience a different workflow. However all commence with an assessment of symptoms, physical examination, medical and familial history. In the United Kingdom CA125 testing is performed prior to ultrasound; in Australia/NZ both are typically performed; and in the US ultrasound is generally performed before biomarker testing. In all cases the information is used to provide the ultimate surgical referral. Specific guidelines (NICE [5], RCOG [48], ACOG [4]) according to region are indicated. Adapted from [49].

Pre-operative discrimination of benign from malignant adnexal masses is critical in the early patient management pathway, where rapid triage to an appropriate specialist has direct correlation with patient outcomes [20]. Whilst standard clinical workflows differ internationally (Figure 2), radiological imaging (typically transvaginal ultrasound) forms the cornerstone of practice designed to identify or exclude a diagnosis of malignancy [4]. Most broadly used for the interpretation of ultrasound imaging are RMI score (combining ultrasound score, CA125 and menopausal status) and the IOTA rules, developed to provide guidance for the differentiation of benign from malignant disease and adapted in several variations including Assessment of Different NEoplasias in the adneXa (ADNEX) and the Ovarian-Adnexal Imaging-Reporting-Data System (O-RADS) system [44,45,46,47]. Patients in whom the risk of malignancy is deemed low can be managed conservatively, either expectantly or laparoscopy; whilst those at high risk can be rapidly referred to specialist oncology surgeons for optimal management [20,21].

The complication that arises in all clinical workflows, however, is where imaging suggests an “intermediate” or undetermined risk of malignancy. In this case CA125 provides little additive benefit [25,28] and does not contribute sufficient diagnostic power to differentiate benign from malignant disease; for example, at a specificity of 90%, RMI provides sensitivity of 72.3% or 64% for identification of malignant disease in post- or pre-menopausal patients, respectively [24]. Similarly, scoring using the IOTA simple rules typically results in ~20% of patients classified as “inconclusive” [26]. In our recent work [30], amongst 169 patients for whom RMI could be calculated were 73 with RMI score >200 suggesting malignancy; however, 27% of these (20 of 73 patients) were subsequently diagnosed with benign disease [30]. Improved biomarker testing is thus required to assist in pre-surgical triage and streamline the referral process. Such testing must provide additive efficacy to ultrasound; and must be able to return results within a short timeframe (typically 24 h) to ensure clinical relevance. Using our multi-marker panel 16 of 20 patients with RMI > 200 indicating malignancy were correctly re-assigned as benign; whilst 2 of 3 with RMI < 200 were correctly assigned as cancers using the panel. Our data suggest that the application of such a panel in the pre-surgical window could significantly improve surgical referral accuracy over CA125 alone.

Correct triage of cases is an essential component of an improved clinical workflow to treat patients with an adnexal mass, both to improve outcomes for all patients as well as reduce burden on the healthcare system. The current dataset is comparatively small hence provides an indication of reclassification performance only. Further evaluation is now required in larger prospective trials aimed at determining the rescue capacity for patients in whom CA125 provides inaccurate or misleading diagnostic information.

## 5. Conclusions

Reclassification analysis demonstrated improved discrimination of benign from malignant ovarian disease using a multi-marker panel compared to CA125, and was effective in both pre- and post-menopausal patient samples. This multi-marker panel may provide an important new tool to assist in the pre-surgical discrimination of patients with suspected ovarian cancer.

## 6. Patents

Aspects of this study are covered by granted patent 2020404453 and provisional patent 540674PRV.

## Figures and Tables

**Table 1:** Summary of cohort details included in this study.

Total Participants (*n*=)		All Samples		Pre-Menopausal		Post-Menopausal	
		334		115		219	
	FIGO Stage	# Cases (*n*=)	CA125 (Median, IQ Range)	# Cases (*n*=)	CA125 (Median, IQ Range)	# Cases (*n*=)	CA125 (Median, IQ Range)
Malignant	all	164	724 (213, 1734)	32	549 (110, 1350)	132	797 (259, 1845)
	stage I	17	178 (62, 527)	11	229 (71, 448)	6	137 (35, 646)
	stages II–IV	147	821 (281, 2105)	21	911 (130, 2743)	126	817 (301, 1988)
Benign	n/a	170	13 (7, 25)	83	14 (8, 26)	87	12 (6, 24)

**Table 2:** Re-classification matrix for CA125 versus Multi-Marker Panel index in pre- or post-menopausal women. Net re-classification index (NRI) was calculated for all samples classified by MMPI at each CA125 threshold (post-menopausal women at 35 U/mL; pre-menopausal women at 67 U/mL, 200 U/mL, or 250 U/mL). Up or down indicate re-classification to higher risk (malignant) or lower risk (benign) groups, respectively.

Combined Risk Score											
	Malignant					Benign					
Menopausal Status	Risk Score CA125	MMP Index ≤ 3.48	MMP Index > 3.48	Up	Down	MMP Index ≤ 3.48	MMP Index > 3.48	Up	Down	NRI (95% CI)	*p* Value
post-menopausal	≤35 U/mL	2	5	0.04	0	72	1	0.011	0.09	0.12 (0.04, 0.21)	0.01
	>35 U/mL	0	125			8	6				
pre-menopausal	≤67 U/mL	4	2	0.06	0.063	72	1	0.012	0.11	0.10 (−0.07, 0.27)	0.26
	>67 U/mL	2	24			9	1				
	≤200 U/mL	5	6	0.18	0.03	78	1	0.012	0.05	0.19 (0.02, 0.37)	0.03
	>200 U/mL	1	21			4	1				
	≤250 U/mL	6	7	0.22	0	79	1	0.01	0.02	0.23 (0.07, 0.41)	0.01
	>250 U/mL	0	19			2	1				

**Table 3:** Number of mis-classifications according to CA125 (U/mL) in pre- and post-menopausal women. The numbers of false negative (cancer with CA125 ≤ threshold) and false positive (benign with CA125 > threshold) findings were compared following re-classification. Total percentage of mis-classifications were calculated according to numbers within pre- or post-menopausal groups, respectively.

	Post-Menopausal		Pre-Menopausal					
	CA125 Threshold 35 U/mL		CA125 Threshold 67 U/mL		CA125 Threshold 200 U/mL		CA125 Threshold 250 U/mL	
	*n*=	misclassified %	*n*=	misclassified %	*n*=	misclassified %	*n*=	misclassified %
total patients	80		80		90		95	
# cancer misclassified CA125	7	8.8%	6	7.5%	11	12.2%	13	13.7%
# cancer misclassified MMPI	2	2.5%	4	5.0%	5	5.6%	6	6.3%
%Δ false negatives	71.4%		66.7%		54.5%		53.8%	
total patients	139		36		25		22	
# benign mis-classified CA125	14	10.1%	10	27.8%	4	16.0%	3	13.6%
# benign mis-classified MMPI	6	4.3%	1	2.8%	1	4.0%	1	4.5%
%D false positives	57.1%		90.0%		75.0%		66.7%	

## Data Availability

The datasets used and/or analyzed during the current study are available from the corresponding author upon reasonable request.

## Supplementary Materials

The following supporting information can be downloaded at: https://www.mdpi.com/article/10.3390/diagnostics14070671/s1, Supplementary Data S1: Diagnosis vs predicted status for all false positive /negative findings by CA125.

## References

[1] Cabasag C.J., Fagan P.J., Ferlay J., Vignat J., Laversanne M., Liu L., van der Aa M.A., Bray F., Soerjomataram I. (2022). Ovarian cancer today and tomorrow: A global assessment by world region and Human Development Index using GLOBOCAN 2020. Int. J. Cancer.

[2] Henderson J.T., Webber E.M., Sawaya G.F. (2018). Screening for Ovarian Cancer: Updated Evidence Report and Systematic Review for the US Preventive Services Task Force. JAMA.

[3] Yeoh M. (2015). Investigation and management of an ovarian mass. Aust. Fam. Physician.

[4] The American College of Obstetricians and Gynecologists, Committee on Practice Bulletins—Gynecology (2016). Practice Bulletin No. 174: Evaluation and Management of Adnexal Masses. Obstet. Gynecol..

[5] National Institute for Health and Care Excellence (2011). Ovarian Cancer: The Recognition and Initial Management of Ovarian Cancer.

[6] Dearking A.C., Aletti G.D., McGree M.E., Weaver A.L., Sommerfield M.K., Cliby W.A. (2007). How relevant are ACOG and SGO guidelines for referral of adnexal mass?. Obstet. Gynecol..

[7] Skates S.J., Mai P., Horick N.K., Piedmonte M., Drescher C.W., Isaacs C., Armstrong D.K., Buys S.S., Rodriguez G.C., Horowitz I.R. (2011). Large prospective study of ovarian cancer screening in high-risk women: CA125 cut-point defined by menopausal status. Cancer Prev. Res..

[8] Sopik V., Rosen B., Giannakeas V., Narod S.A. (2015). Why have ovarian cancer mortality rates declined? Part III. Prospects for the future. Gynecol. Oncol..

[9] Nustad K., Bast R.C., Brien T.J., Nilsson O., Seguin P., Suresh M.R., Saga T., Nozawa S., Bormer O.P., de Bruijn H.W. (1996). Specificity and affinity of 26 monoclonal antibodies against the CA 125 antigen: First report from the ISOBM TD-1 workshop. International Society for Oncodevelopmental Biology and Medicine. Tumour Biol..

[10] Carney M.E., Lancaster J.M., Ford C., Tsodikov A., Wiggins C.L. (2002). A population-based study of patterns of care for ovarian cancer: Who is seen by a gynecologic oncologist and who is not?. Gynecol. Oncol..

[11] Earle C.C., Schrag D., Neville B.A., Yabroff K.R., Topor M., Fahey A., Trimble E.L., Bodurka D.C., Bristow R.E., Carney M. (2006). Effect of surgeon specialty on processes of care and outcomes for ovarian cancer patients. J. Natl. Cancer Inst..

[12] Burgess B.T., Ueland F.R. (2019). Adnexal tumors in menopausal women: Surgery or surveillance?. Menopause.

[13] Donnez J. (2018). Fertility preservation in women, focusing on cancer, benign diseases and social reasons. Minerva Ginecol..

[14] Oktay K., Harvey B.E., Partridge A.H., Quinn G.P., Reinecke J., Taylor H.S., Wallace W.H., Wang E.T., Loren A.W. (2018). Fertility Preservation in Patients With Cancer: ASCO Clinical Practice Guideline Update. J. Clin. Oncol..

[15] Schuring A.N., Fehm T., Behringer K., Goeckenjan M., Wimberger P., Henes M., Henes J., Fey M.F., von Wolff M. (2018). Practical recommendations for fertility preservation in women by the FertiPROTEKT network. Part I: Indications for fertility preservation. Arch. Gynecol. Obstet..

[16] Liu D., Cai J., Gao A., Wang Z., Cai L. (2020). Fertility sparing surgery vs radical surgery for epithelial ovarian cancer: A meta-analysis of overall survival and disease-free survival. BMC Cancer.

[17] Chan J.K., Kapp D.S., Shin J.Y., Husain A., Teng N.N., Berek J.S., Osann K., Leiserowitz G.S., Cress R.D., O’Malley C. (2007). Influence of the gynecologic oncologist on the survival of ovarian cancer patients. Obstet. Gynecol..

[18] Engelen M.J., Kos H.E., Willemse P.H., Aalders J.G., de Vries E.G., Schaapveld M., Otter R., van der Zee A.G. (2006). Surgery by consultant gynecologic oncologists improves survival in patients with ovarian carcinoma. Cancer.

[19] Giede K.C., Kieser K., Dodge J., Rosen B. (2005). Who should operate on patients with ovarian cancer? An evidence-based review. Gynecol. Oncol..

[20] Vernooij F., Heintz P., Witteveen E., van der Graaf Y. (2007). The outcomes of ovarian cancer treatment are better when provided by gynecologic oncologists and in specialized hospitals: A systematic review. Gynecol. Oncol..

[21] Canis M., Rabischong B., Houlle C., Botchorishvili R., Jardon K., Safi A., Wattiez A., Mage G., Pouly J.L., Bruhat M.A. (2002). Laparoscopic management of adnexal masses: A gold standard?. Curr. Opin. Obstet. Gynecol..

[22] Funston G., Van Melle M., Baun M.L., Jensen H., Helsper C., Emery J., Crosbie E.J., Thompson M., Hamilton W., Walter F.M. (2019). Variation in the initial assessment and investigation for ovarian cancer in symptomatic women: A systematic review of international guidelines. BMC Cancer.

[23] Friedrich L., Meyer R., Levin G. (2021). Management of adnexal mass: A comparison of five national guidelines. Eur. J. Obstet. Gynecol. Reprod. Biol..

[24] Davenport C.F., Rai N., Sharma P., Deeks J., Berhane S., Mallett S., Saha P., Solanki R., Bayliss S., Snell K. (2022). Diagnostic Models Combining Clinical Information, Ultrasound and Biochemical Markers for Ovarian Cancer: Cochrane Systematic Review and Meta-Analysis. Cancers.

[25] Ashmore A.A., Gnanachandran C., Luqman I., Horrocks K. (2021). One-stop clinic for patients with suspected ovarian cancer: Results from a retrospective outcome study of the referral pathway. BMC Womens Health.

[26] Auekitrungrueng R., Tinnangwattana D., Tantipalakorn C., Charoenratana C., Lerthiranwong T., Wanapirak C., Tongsong T. (2019). Comparison of the diagnostic accuracy of International Ovarian Tumor Analysis simple rules and the risk of malignancy index to discriminate between benign and malignant adnexal masses. Int. J. Gynaecol. Obstet..

[27] Expert Panel on Women’s I., Atri M., Alabousi A., Reinhold C., Akin E.A., Benson C.B., Bhosale P.R., Kang S.K., Lakhman Y., Nicola R. (2019). ACR Appropriateness Criteria^®^ Clinically Suspected Adnexal Mass, No Acute Symptoms. J. Am. Coll. Radiol..

[28] Pelayo M., Pelayo-Delgado I., Sancho-Sauco J., Sanchez-Zurdo J., Abarca-Martinez L., Corraliza-Galan V., Martin-Gromaz C., Pablos-Antona M.J., Zurita-Calvo J., Alcazar J.L. (2023). Comparison of Ultrasound Scores in Differentiating between Benign and Malignant Adnexal Masses. Diagnostics.

[29] Kang S.W., Rainczuk A., Oehler M.K., Jobling T.W., Plebanski M., Stephens A.N. (2021). Active Ratio Test (ART) as a Novel Diagnostic for Ovarian Cancer. Diagnostics.

[30] Stephens A.N., Hobbs S.J., Kang S.W., Bilandzic M., Rainczuk A., Oehler M.K., Jobling T.W., Plebanski M., Allman R. (2023). A Novel Predictive Multi-Marker Test for the Pre-Surgical Identification of Ovarian Cancer. Cancers.

[31] Cook N.R. (2018). Quantifying the added value of new biomarkers: How and how not. Diagn. Progn. Res..

[32] Dite G.S., MacInnis R.J., Bickerstaffe A., Dowty J.G., Allman R., Apicella C., Milne R.L., Tsimiklis H., Phillips K.A., Giles G.G. (2016). Breast Cancer Risk Prediction Using Clinical Models and 77 Independent Risk-Associated SNPs for Women Aged Under 50 Years: Australian Breast Cancer Family Registry. Cancer Epidemiol. Biomarkers Prev..

[33] Barrett J., Jenkins V., Farewell V., Menon U., Jacobs I., Kilkerr J., Ryan A., Langridge C., Fallowfield L., Trialists U. (2014). Psychological morbidity associated with ovarian cancer screening: Results from more than 23,000 women in the randomised trial of ovarian cancer screening (UKCTOCS). BJOG.

[34] Andersen M.R., Drescher C.W., Zheng Y., Bowen D.J., Wilson S., Young A., McIntosh M., Mahony B.S., Lowe K.A., Urban N. (2007). Changes in cancer worry associated with participation in ovarian cancer screening. Psychooncology.

[35] Hassan H., Allen I., Sofianopoulou E., Walburga Y., Turnbull C., Eccles D.M., Tischkowitz M., Pharoah P., Antoniou A.C. (2023). Long-term outcomes of hysterectomy with bilateral salpingo-oophorectomy: A systematic review and meta-analysis. Am. J. Obstet. Gynecol..

[36] Parker W.H., Jacoby V., Shoupe D., Rocca W. (2009). Effect of bilateral oophorectomy on women’s long-term health. Women’s Health.

[37] Gottschau M., Rosthoj S., Settnes A., Aalborg G.L., Viuff J.H., Munk C., Jensen A., Kjaer S.K., Mellemkjaer L. (2023). Long-Term Health Consequences After Ovarian Removal at Benign Hysterectomy: A Nationwide Cohort Study. Ann. Intern. Med..

[38] American College of Obstetricians and Gynecologists (2007). ACOG Practice Bulletin. Management of adnexal masses. Obstet. Gynecol..

[39] Hall T.R., Randall T.C. (2015). Adnexal masses in the premenopausal patient. Clin. Obstet. Gynecol..

[40] Whiteman M.K., Kuklina E., Jamieson D.J., Hillis S.D., Marchbanks P.A. (2010). Inpatient hospitalization for gynecologic disorders in the United States. Am. J. Obstet. Gynecol..

[41] Bolstad N., Oijordsbakken M., Nustad K., Bjerner J. (2012). Human epididymis protein 4 reference limits and natural variation in a Nordic reference population. Tumour Biol..

[42] Schorge J.O., Eisenhauer E.E., Chi D.S. (2012). Current surgical management of ovarian cancer. Hematol. Oncol. Clin. N. Am..

[43] Dunton C.J., Hutchcraft M.L., Bullock R.G., Northrop L.E., Ueland F.R. (2021). Salvaging Detection of Early-Stage Ovarian Malignancies When CA125 Is Not Informative. Diagnostics.

[44] Vara J., Manzour N., Chacon E., Lopez-Picazo A., Linares M., Pascual M.A., Guerriero S., Alcazar J.L. (2022). Ovarian Adnexal Reporting Data System (O-RADS) for Classifying Adnexal Masses: A Systematic Review and Meta-Analysis. Cancers.

[45] Van Calster B., Van Hoorde K., Valentin L., Testa A.C., Fischerova D., Van Holsbeke C., Savelli L., Franchi D., Epstein E., Kaijser J. (2014). Evaluating the risk of ovarian cancer before surgery using the ADNEX model to differentiate between benign, borderline, early and advanced stage invasive, and secondary metastatic tumours: Prospective multicentre diagnostic study. BMJ.

[46] Timmerman D., Van Calster B., Testa A., Savelli L., Fischerova D., Froyman W., Wynants L., Van Holsbeke C., Epstein E., Franchi D. (2016). Predicting the risk of malignancy in adnexal masses based on the Simple Rules from the International Ovarian Tumor Analysis group. Am. J. Obstet. Gynecol..

[47] Timmerman D., Testa A.C., Bourne T., Ferrazzi E., Ameye L., Konstantinovic M.L., Van Calster B., Collins W.P., Vergote I., Van Huffel S. (2005). Logistic regression model to distinguish between the benign and malignant adnexal mass before surgery: A multicenter study by the International Ovarian Tumor Analysis Group. J. Clin. Oncol..

[48] (2011). Green–top Guideline No. 62. Management of Suspected Ovarian Masses in Premenopausal Women. RCOG/BSGE Joint Guideline.

[49] Bullock B., Larkin L., Turker L., Stampler K. (2022). Management of the Adnexal Mass: Considerations for the Family Medicine Physician. Front. Med..

