# Supplementary material for: ReClassification of Patients with Ambiguous CA125 for Optimised Pre-Surgical Triage
Source: Diagnostics (Basel). 2024 Mar 22;14(7):671. doi: 10.3390/diagnostics14070671 (PMC11011550; doi:10.3390/diagnostics14070671)
Supplement: Supplementary file 1 [file diagnostics-14-00671-s001.zip › diagnostics-2860188-supplementary.pdf]

Supplementary Data S1. Diagnosis *vs* predicted status for all false positive / negative findings by CA125. Predicted *vs* actual diagnosis and pathology information for each sample mis-diagnosed by CA125. (A) post-menopausal, and (B) pre-menopausal patients.

**A. Post-menopausal patients**								**Predicted malignant (1=yes, 0=no)**	
**Sample Identifier**	**menopausal status**	**Diagnosis**	**Pathology**	**Grade**	**Stage**	**Pre-OP CA125**	**MMP Index**	**CA125 ≥35U/ml**	**MMP Index ≥3.648**
**66**	post	Malignant	serous	1	3C	8	1.853	0	0
**526**	post	Malignant	endometroid	3	1A	9	7.501	0	1
**999**	post	Malignant	mucinous	1	1A	14	2.091	0	0
**452**	post	Malignant	serous papillary/some squamoid appearance	3	3C	20	7.007	0	1
**976**	post	Malignant	serous	3	3C	21.3	8.775	0	1
**986**	post	Malignant	serous	3	3C	23.8	8.784	0	1
**33**	post	Malignant	serous	2	3C	34	6.091	0	1
**26**	post	Benign	cystadenoma	n/a	n/a	35	0.715	1	0
**EP0392**	post	Benign	simple cyst	n/a	n/a	35	0.898	1	0
**140**	post	Benign	mucinous fibroma & Brenner tumour	n/a	n/a	35	3.451	1	0
**1082**	post	Benign	serous adenofibroma& Brenner tumour	n/a	n/a	35.1	1.784	1	0
**620**	post	Benign	fibrothecoma	n/a	n/a	39	7.209	1	1
**753**	post	Benign	cystadenoma	n/a	n/a	43	0.096	1	0
**646**	post	Benign	fibroma	n/a	n/a	56	3.158	1	0
**922**	post	Benign	fibroma	n/a	n/a	63	1.333	1	0
**560**	post	Benign	fibroma	n/a	n/a	71	0.582	1	0
**98**	post	Benign	simple cyst	n/a	n/a	102	2.155	1	0
**47**	post	Benign	cystadenoma	n/a	n/a	146	4.084	1	1
**352**	post	Benign	Haemorrhagic ovarian torsion	n/a	n/a	179	8.880	1	1
**788**	post	Benign	fibroma	n/a	n/a	268	1.394	1	0
**775**	post	Benign	Papillary adenofibroma	n/a	n/a	274	0.897	1	0
**615**	post	Benign	cystadenoma	n/a	n/a	282.5	9.692	1	1
**843**	post	Benign	cystadenofibroma	n/a	n/a	345	9.252	1	1
**512**	post	Benign	simple cyst	n/a	n/a	767	9.102	1	1

B. Pre-menopausal patients								Predicted malignant (1=yes, 0=no)			
**Sample Identifier**	**menopausal status**	**Diagnosis**	**Pathology**	**Grade**	**Stage**	**Pre-OP CA125**	**MMP Index**	**CA125 ≥67U/ml**	**CA125 ≥200U/ml**	**CA125 ≥250U/ml**	**MMP Index ≥3.648**
**343**	pre	Benign	fibroma	n/a	n/a	1841	0.128	1	1	1	0
**845**	pre	Malignant	mucinous	2	1A	15	0.173	0	0	0	0
**388**	pre	Malignant	Granulosa cell tumour	2	1A	10	0.244	0	0	0	0
**287**	pre	Benign	Xanthogranulomatous	n/a	n/a	538	0.377	1	1	1	0
**1016**	pre	Malignant	serous	3	3B	25	0.755	0	0	0	0
**450**	pre	Malignant	serous	2	3B	130	1.545	1	0	0	0
**847**	pre	Benign	Lipoid cell tumour	n/a	n/a	245	3.249	1	1	0	0
**301**	pre	Malignant	serous / clear cell	3	3C	58	3.684	0	0	0	1
**649**	pre	Malignant	dysgerminoma	2	1B	80	4.583	1	0	0	1
**56**	pre	Malignant	mucinous	1	1C	62	5.324	0	0	0	1
**30**	pre	Benign	cystadenomata	n/a	n/a	546	5.569	1	1	1	1
**136**	pre	Malignant	clear cell	3	1C	132	7.825	1	0	0	1
**336**	pre	Malignant	serous	2	3C	120	9.393	1	0	0	1
**147**	pre	Malignant	serous	3	3C	40	9.537	0	0	0	1
**317**	pre	Malignant	serous	3	3C	80	9.967	1	0	0	1
